# Neonatal intubation: what are we doing?

**DOI:** 10.1007/s00431-023-05418-x

**Published:** 2024-01-23

**Authors:** Sabina Maglio, Francesco Cavallin, Chiara Sala, Benedetta Bua, Paolo Ernesto Villani, Arianna Menciassi, Selene Tognarelli, Daniele Trevisanuto

**Affiliations:** 1 The BioRobotics Institute, Scuola Superiore Sant’Anna, Pisa, Italy; 2 Department of Excellence in Robotics & AI, Scuola Superiore Sant’Anna, Pisa, Italy; 3 Independent Statistician, Solagna, Italy; 4 Department of Pediatric Anesthesia and Intensive Care “V. Buzzi” Children’s Hospital, University of Milan, Milan, Italy; 5 Department of Women and Children Health, University Hospital of Padua, Via Giustiniani, 3, 35128 Padua, Italy; 6 Department of Woman’s and Child’s Health, Poliambulanza Hospital, Fondazione Poliambulanza, Brescia, Italy

**Keywords:** Forces, Intubation, Neonate

## Abstract

How and when the forces are applied during neonatal intubation are currently unknown. This study investigated the pattern of the applied forces by using sensorized laryngoscopes during the intubation process in a neonatal manikin. Nine users of direct laryngoscope and nine users of straight-blade video laryngoscope were included in a neonatal manikin study. During each procedure, relevant forces were measured using a force epiglottis sensor that was placed on the distal surface of the blade. The pattern of the applied forces could be divided into three sections. With the direct laryngoscope, the first section showed either a quick rise of the force or a discontinuous rise with several peaks; after reaching the maximum force, there was a sort of plateau followed by a quick drop of the applied forces. With the video laryngoscope, the first section showed a quick rise of the force; after reaching the maximum force, there was an irregular and heterogeneous plateau, followed by heterogeneous decreases of the applied forces. Moreover, less forces were recorded when using the video laryngoscope.

*    Conclusions*: This neonatal manikin study identified three sections in the diagram of the forces applied during intubation, which likely mirrored the three main phases of intubation. Overall, the pattern of each section showed some differences in relation to the laryngoscope (direct or video) that was used during the procedure. These findings may provide useful insights for improving the understanding of the procedure.

**Table:** 

**What is Known:** *• Neonatal intubation is a life-saving procedure that requires a skilled operator and may cause direct trauma to the tissues and precipitate adverse reactions.* *• Intubation with a videolaryngoscope requires less force than with a direct laryngoscope, but how and when the forces are applied during the whole neonatal intubation procedure are currently unknown.*	
**What is New:** *• Forces applied to the epiglottis during intubation can be divided into three sections: (i) an initial increase, (ii) a sort of plateau, and (iii) a decrease.* *• The pattern of each section shows some differences in relation to the laryngoscope (direct or videolaryngoscope) that is used during the procedure.*

**Supplementary Information:**

The online version contains supplementary material available at 10.1007/s00431-023-05418-x.

## Introduction

Neonatal intubation is a life-saving procedure that requires a skilled operator. However, health care providers have been progressively less exposed to such procedure over time due to changes in clinical practice [1, 2]. While neonatal training programs should provide suitable opportunities to learn this skill, a deep understanding about how the procedure is performed may offer useful information to trainees and trainers in order to achieve adequate proficiency [3].

**Fig. 1:**
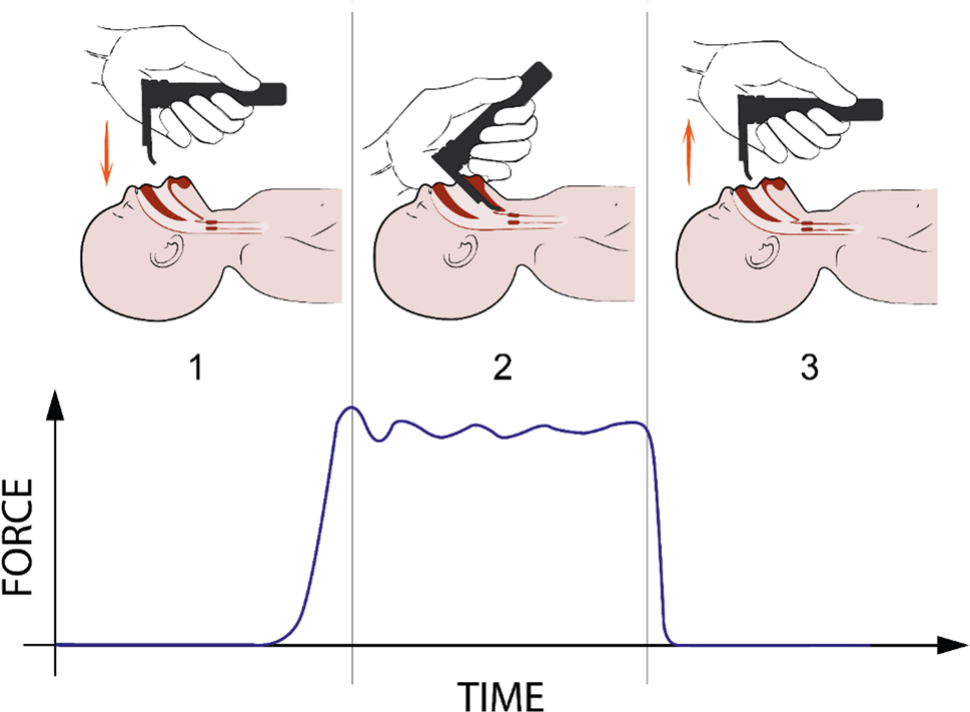
Intubation procedure: schematic representation of the three different phases as described in the text, i.e., 1. insertion of the laryngoscope, 2. alignment of the soft structures and tube insertion, 3. laryngoscope removal. A typical force trend is reported directly under the schematic representation in order to discriminate the force acquisition during the entire procedure

Neonatal intubation procedure consists of three main phases: (1) insertion of the laryngoscope, (2) alignment of the soft structures with tube insertion for vocal cords visualization, and (3) laryngoscope removal leaving the tube in the right position [4]. During the insertion, the laryngoscope slides gently over the tongue and its tip reaches the larynx. In this phase, the interactions between the tool and the soft tissues are either inexistent or so slight to generate a detectable force on the soft tissues. On the contrary, in the second phase, the laryngoscope is pushed against the oro-tracheal tissues to align them and guarantee the right point of view for the tube insertion. This movement generates a compression force on the baby tissues that can be dangerous in terms of tissue ischemia or perforation, or future problems in the development of the surrounding structures [5, 6]. Additionally, the compression forces generated during the alignment phase are maintained high also during the tube insertion, when the soft tissues have to be lifted to allow the vocal cord visualization. A significant force is thus generated and maintained high for a long time, i.e., until 30 s [7]. Finally, once the tube is in the right position, the laryngoscope is pulled out of the mouth and the pressure against the tissues is removed (Fig. 1). In order to evaluate the forces induced on the soft tissues, the same authors have previously developed sensorized video laryngoscopes which were properly tested in neonatal simulated environments, aiming at guaranteeing a safety intubation procedure [8]. However, how and when the forces are applied during the whole neonatal intubation procedure are currently unknown. This study aimed to investigate the pattern of the forces recorded by the sensors integrated on the laryngoscope during the whole intubation process in a neonatal manikin.

## Methods

### Study design

This is an observational sub-study from a previous trial [9] describing how the applied forces vary during the whole intubation process using a neonatal manikin. The original trial was conducted at the University of Padua Hospital (Italy) in January 2022. Being a simulation study on manikin, no formal ethical approval was required by the Ethics Committee of the University Hospital of Padua (prot.n.0002658). Written informed consent was obtained from participants.

### Procedures

Details about trial procedures have been previously reported [9]. Briefly, level III neonatal and pediatric intensive care unit clinicians, pediatric residents, anesthesiology clinicians, and anesthesiology residents of the University of Padua randomly performed intubations on the NewBorn Anne neonatal simulator (Laerdal, Stavanger, Norway) using a direct laryngoscope and two models of video laryngoscope (straight-blade video laryngoscope and hyper-angulated blade video laryngoscope).

In this sub-study, we evaluated the data that were recorded during attempts by more experienced users (clinicians with 10 or more intubations on patient) with a successful intubation in less than 30 s. Since there were no experienced users for the hyper-angulated blade video laryngoscope, the analysis was restricted to the forces applied with the standard direct laryngoscope with size 1 Miller blade and the video laryngoscope, the Verathon GlideScope Core, with Miller blade Spectrum S1 (Verathon Inc., USA). A not cuffed, 3-mm size endotracheal tube (ETT) was used for each intubation.

### Force measurements

**Fig. 2:**
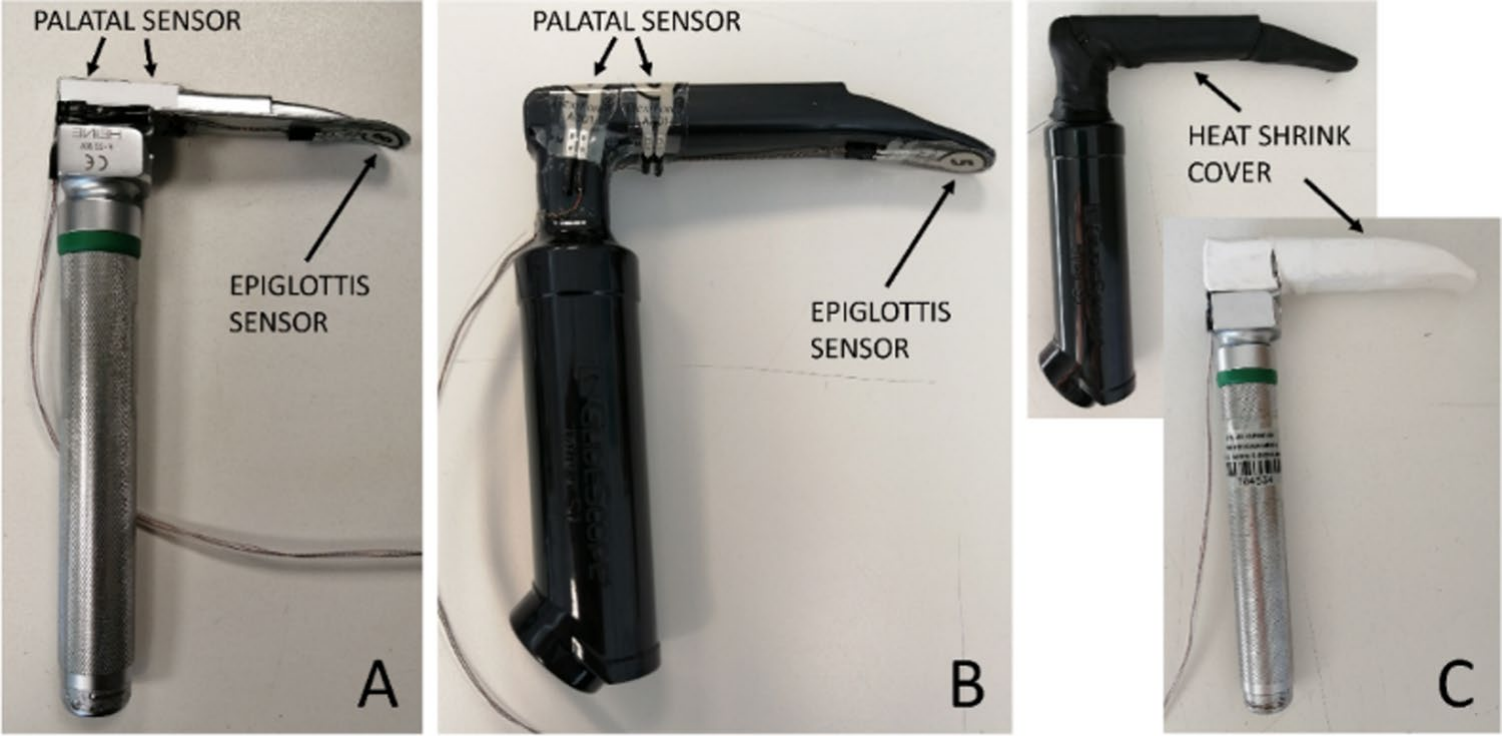
Position of epiglottis sensor and palatal sensors in direct laryngoscope (**A**), and in video laryngoscope (**B**). Heat shrinks cover position (**C**)

During each procedure, force measurements were acquired using three commercial force sensors FlexiForce A301 (Tekscan Inc., MA, USA) fixed through a double-sided tape in three different sites of each blade, previously identified as the most significant for the intubation forces recording. One sensor, i.e., the epiglottis sensor, was placed on the distal surface of the blade in correspondence to the instrument area that should come in contact with the epiglottis during the intubation and the other two sensors, i.e., palatal sensors, were firmly attached on the proximal surface of the blade at the area in touch withing the upper gum and the hard palate (Fig. 2A and B). The whole blade surface was then covered with heat-sink tubes in order to hide the sensor positions to the study participants and protect the sensors during the procedure (Fig. 2C). Each force sensor had a sensing area of 9.53-mm diameter, thickness of 0.203 mm, response time of < 5 μs, and a linearity error of ± 3% and a force range (0–111 N) suitable for the described application. The output signal from the sensors was conditioned and amplified with the electronic components suggested in the sensors data sheet and acquired by means of an Arduino Uno board (Arduino, Italy). Using LabVIEW software (National Instruments, TX, USA), data sensors were individually filtered to remove part of the noise and saved in a text file. Sensor calibration curves were extrapolated from bench tests and integrated in the LabVIEW scripts, one for each laryngoscope. Finally, a graphical user interface (GUI) for real-time data visualization was implemented. Data saving started few seconds before the procedure begins, i.e., when the laryngoscope was introduced into the mouth and ended few seconds after the procedures end, i.e., when the laryngoscope was pulled out of the mouth. The selected Arduino board and its interface with LabVIEW software guaranteed an adequate sensor signal sampling for this application.

### Data collection

In order to use the recorded force trends for defining and describing the different intubation phases, the measures of interest were the contact forces between the sensorized instruments and soft tissues. Only epiglottis sensors were used as reference for force investigation because the right intubation procedure generates forces only on the distal surface of the blade (Fig. 1). In fact, any other forces induced on the tongue and palate were associated with an improper procedure; thus, the data were excluded from the analysis. All data were collected by an external clinical observer who was not involved in the simulation. Data were recorded on a data sheet designed for the study and stored in a password-protected computer.

### Data analysis

**Fig. 3:**
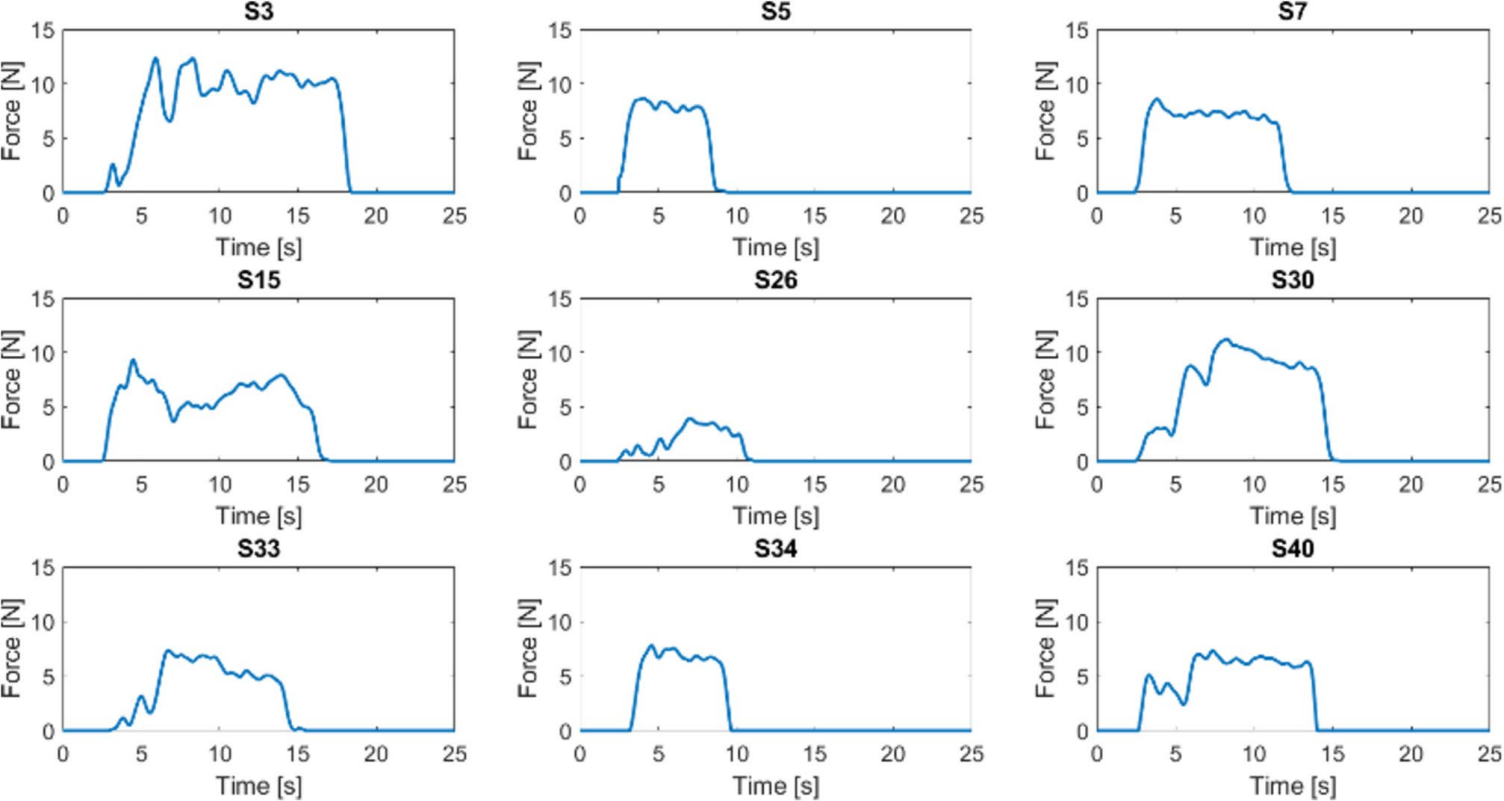
Pressure forces registered by the epiglottis sensor during each selected attempt by using the direct laryngoscope. Forces are presented as one diagram per subject (S) with the time (seconds) plotted on the x-axis and the force amplitude, expressed in Newton, on the y-axis

**Fig. 4:**
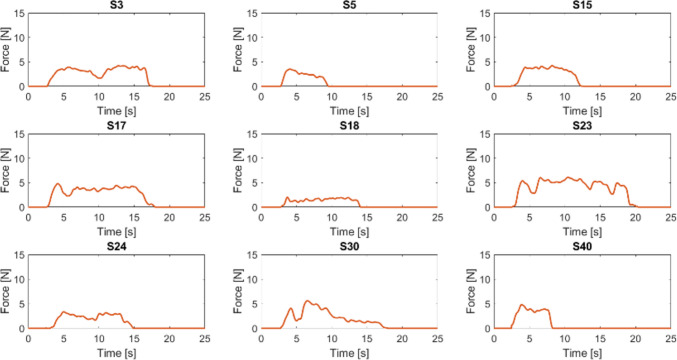
Forces registered by the epiglottis sensor during each selected attempt by using the video laryngoscope. Forces are presented as one diagram per subject (S) with the time (seconds) plotted on the x-axis and the amplitude of the force, expressed in Newton, on the y-axis

Data analysis was performed with MATLAB software (MathWorks, CA, USA). Data not concerning the intubation phase were manually discarded. The noise coming from the sensors was removed by means of a 20-sample mobile mean filter and negative values were substituted with a zero—negative values have no physical meaning and come from the remaining noise. The resulting data were plotted in a graph where the force value expressed in Newton belongs on the y-axis and the time in seconds belongs on the x-axis. The number of samples was directly proportional with the intubation time and the final amounts were due to the cumulative sampling frequency (40 samples per second) imposed by the electronics together with the acquisition software features. The x-axis of all the graphs covered a 0 to 1000 sample interval, corresponding approximately to 25 s, in order to display the acquired force values for the whole procedure and to streamline the visual comparison between the recorded curves. Each selected attempt was plotted in its own graph and then properly grouped by used blade (Fig. 3 for direct laryngoscope and Fig. 4 for the videolaryngoscope). To allow further analysis, both the standard laryngoscope and the videolaryngoscopes attempts for the same subject were included into a comprehensive plot (Supplementary Fig. 1).

## Results

Among the 40 participants in the original trial, the analysis included the recordings from nine clinicians with 10 or more intubations on patients with direct laryngoscope (subjects 3, 5, 7, 15, 26, 30, 33, 34, 40) and clinicians with 10 or more intubations on patients with straight-blade video laryngoscope (subjects 3, 5, 15, 17, 18, 23, 24, 30, 40).

The trend of the forces recorded by the epiglottis sensor is shown, divided by subject (S), in Figs. 3 and 4 for the direct and for the video laryngoscope, respectively. The discussion of these diagrams is presented in the next section.

In five subjects who had experience with both laryngoscopes, we plotted the trend of the forces applied when using the devices in Supplementary Fig. 1. The curves are aligned on the x-axis by considering the same starting point for each attempt.

## Discussion

In this study, we investigated how and when health care providers applied forces during the intubation procedure using a neonatal manikin.

**Fig. 5:**
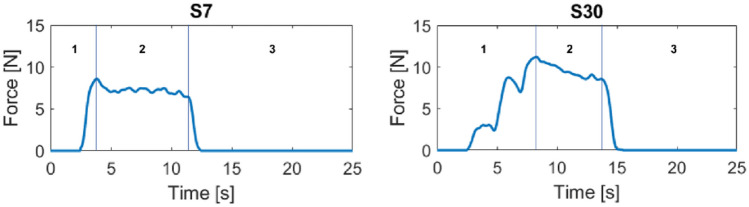
Force diagrams of subjects S7 and S30 extracted from Fig. 3, as example for the identification of the three sections of the diagram

Overall, the recordings of the forces pictured a shape that can be divided in three sections (i) rising of the applied forces, (ii) a sort of plateau, and (iii) a decrease of the applied forces (Fig. 5). This shape likely mirrored the three main phases of intubation including the insertion of the laryngoscope, the alignment of the soft structures with tube insertion for vocal cords visualization, and the removal of the laryngoscope after tube positioning [4].

When the participants used the direct laryngoscope, we could identify two patterns for the first section of the force diagram. The first pattern showed a quite steep slope up to reach the maximum of the plot (as for S7 in Fig. 3), while the second pattern displayed a discontinuous rise with several peaks followed by short downhills (as for S30 in Fig. 3). Based on the clinical experience of the authors, and using the notes taken during the simulations as reference, we could associate the first pattern to participants who quickly find a good vision of the vocal cords, while the second pattern may be explained by a continuous adjustment of the laryngoscope position to achieve the best vision of the vocal cord and thus the correct and safe tube insertion. In addition, the force diagrams with a quite steep slope in the first sections generally showed a shorter time of intubation. After reaching the maximum of the plot, the forces were maintained approximatively constant with small variations, that were compatible with the alignment of the soft structures while inserting the endotracheal tube. This plateau had similar patterns in all participants but two (S3 and S15). Thereafter, there was a quick drop of the applied forces by all participants due to the removal of the laryngoscope.

When the participants used the video laryngoscope, the first section of the force diagram broadly showed a similar pattern for all participants, displaying a quite steep slope up to reach the maximum of the plot. The second section consisted of an irregular and heterogeneous plateau, which was sometimes longer and more irregular when compared to the second section of the plots referring to the procedure with the direct laryngoscope. The third section showed heterogeneous decreases of the applied forces that were mostly less steep compared to the third section of the plots referring to the procedure with the direct laryngoscope.

The indirect comparison between the procedures performed using the two laryngoscopes may have the following interpretations. The first section of the force diagram may suggest that the video laryngoscope allowed a better vision of the vocal cords, as reported in literature [10]. The second section may suggest that the video laryngoscope sometimes required more adjustments to maintain a proper vision of the vocal cords and more time to insert the tube. While the longer time for tube insertion has already been reported in literature [10, 11], we can speculate that the participants were more familiar with the direct laryngoscope, despite having a sufficient experience with both devices. The third section may suggest that the participants prolonged the visualization of the glottis on the screen while removing the blade of the video laryngoscope. In addition, our recordings confirmed that less forces were required when using the video laryngoscope, as reported in previous studies [9, 12]. Overall, there was large heterogeneity in terms of applied forces and duration of the procedure among the participants. We suppose that some factors such as experience and sex may influence those aspects, but the small sample size prevented from any reasonable explorations on such topic.

In the five participants who had experience with both direct and videolaryngoscopes, the direct comparison of the forces applied with the two devices broadly confirmed the interpretations that were suggested by the indirect comparison.

To our knowledge, this is the first study describing the pattern of the applied forces when using a laryngoscope during neonatal intubation. While the analysis of the magnitude of the applied forces with different laryngoscopes was outside the scope of this study and has already been addressed elsewhere [9, 12], we believe that our data provide some insights on how and when forces are applied to the soft tissues when using direct and video laryngoscopes. These may be a source of discussion for those involved in neonatal intubation, and may offer useful information to trainees and trainers in order to achieve adequate proficiency.

Our study has some limitations that should be considered when reading the results. First, the small sample size suggests caution in the interpretation of the results. Second, the simulation may limit the generalizability of the findings to the patient because the manikin removes the anatomical variability among neonates. Third, the simulation setting induces a low-stress environment, which may bias the performance of the participants. However, previous studies suggested that the forces used in a manikin were similar to those applied to humans [12, 13]. Last, we acknowledge the arbitrariness of including only clinicians with 10 or more intubations on patients with direct or video laryngoscope, as more experienced users among participants in the original trial [9]. Hence, the generalizability of the findings should be limited to clinicians with similar experience.

## Conclusions

In this neonatal manikin study, we identified three sections in the diagram of the forces applied during intubation, displaying a rising of the forces, followed by a sort of plateau and a final decrease of the forces. Overall, the pattern of each section showed some differences in relation to the laryngoscope (direct or video) that was used during the procedure. These findings may provide useful insights to health care providers involved in neonatal intubation, in order to improve the understanding and the quality of the procedure.

### Supplementary Information

Below is the link to the electronic supplementary material.

Supplementary file1 (PNG 58 KB)

## Data Availability

All data generated or analyzed during this study are included in this article. Further inquiries can be directed to the corresponding author.

## References

[1] Evans P, Shults J, Weinberg DD, Napolitano N, Ades A, Johnston L, Levit O, Brei B, Krick J, Sawyer T, Glass K, Wile M, Hollenberg J, Rumpel J, Moussa A, Verreault A, Abou Mehrem A, Howlett A, McKanna J, Nishisaki A, Foglia EE (2021) Intubation competence during neonatal fellowship training. Pediatrics 148:e2020036145 34172556 10.1542/peds.2020-036145 PMC8290971

[2] Sawyer T, Foglia EE, Ades A et al (2019) National Emergency Airway Registry for Neonates (NEAR4NEOS) Investigators. Incidence, impact and indicators of difficult intubations in the neonatal intensive care unit: a report from the National Emergency Airway Registry for Neonates. Arch Dis Child Fetal Neonatal Ed 104:F461–F466 30796059 10.1136/archdischild-2018-316336

[3] O’Donnell CPF, Kamlin COF, Davis PG, Morley CJ (2006) Endotracheal intubation attempts during neonatal resuscitation: success rates, duration, and adverse effects. Pediatrics 117:e16-21 16396845 10.1542/peds.2005-0901

[4] Goldsmith JP, Karotkin E (1996) Assisted ventilation of the neonate, 3rd Edition, Saunders Company

[5] Hatch LD, Grubb PH, Lea AS et al (2016) Endotracheal intubation in neonates: a prospective study of adverse safety events in 162 infants. J Pediatr 168:62–66 26541424 10.1016/j.jpeds.2015.09.077 PMC4698044

[6] Foglia EE, Ades A, Sawyer T et al (2019) Neonatal intubation practice and outcomes: an international registry study. Pediatrics 143:e20180902 30538147 10.1542/peds.2018-0902 PMC6317557

[7] Weiner GM, editor (2021) Textbook of neonatal resuscitation. American Academy of Pediatrics and American Heart Association; 8th Ed

[8] Selene T, Baldoli I, Scaramuzzo RT, Ciantelli M, Francesca Cecchi M, Gentile CL, Sigali E, Menciassi A, Cuttano A (2014) Development and validation of a sensorized neonatal intubation skill trainer for simulation based education enhancement. IJMRHS 3:833–840

[9] Cavallin F, Sala C, Maglio S, Bua B, Villani PE, Menciassi A, Tognarelli S, Trevisanuto D (2023) Applied forces with direct versus indirect laryngoscopy in neonatal intubation: a randomized crossover mannequin study. Can J Anaesth 70:861–868 36788198 10.1007/s12630-023-02402-9

[10] Abdelgadir IS, Phillips RS, Singh D, Moncreiff MP, Lumsden JL (2017) Videolaryngoscopy versus direct laryngoscopy for tracheal intubation in children (excluding neonates). Cochrane Database Syst Rev 5:CD011413 28539007 10.1002/14651858.CD011413.pub2 PMC6481531

[11] Lingappan K, Neveln N, Arnold JL, Fernandes CJ, Pammi M (2023) Videolaryngoscopy versus direct laryngoscopy for tracheal intubation in neonates. Cochrane Database Syst Rev 5:CD009975 37171122 10.1002/14651858.CD009975.pub4 PMC10177149

[12] Gordon JK, Bertram VE, Cavallin F, Parotto M, Cooper RM (2020) Direct versus indirect laryngoscopy using a Macintosh video laryngoscope: a mannequin study comparing applied forces. Can J Anaesth 67:515–520 32152886 10.1007/s12630-020-01583-x

[13] Lee C, Russell T, Firat M, Cooper RM (2013) Forces generated by Macintosh and GlideScope® laryngoscopes in four airway-training manikins. Anaesthesia 68:492–496 23573844 10.1111/anae.12209

