# Supplementary figures and images for: Neonatal intubation: what are we doing?
Source: Eur J Pediatr. 2024 Jan 23;183(4):1811–7. doi: 10.1007/s00431-023-05418-x (PMC11001655; doi:10.1007/s00431-023-05418-x)

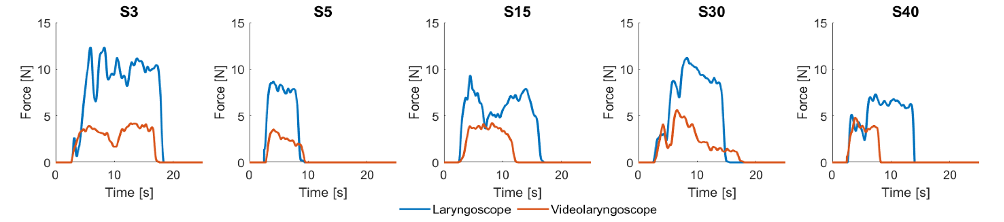

Supplement: Supplementary file 1 — Supplementary file1 (PNG 58 KB) [file 431_2023_5418_MOESM1_ESM.png]
